# Design and Synthesis of New Dual Binding Site Cholinesterase Inhibitors: *in vitro* Inhibition Studies with *in silico* Docking

**DOI:** 10.2174/15701808113106660078

**Published:** 2014-03

**Authors:** Muhammad Yar, Marek Bajda, Rana Atif Mehmood, Lala Rukh Sidra, Nisar Ullah, Lubna Shahzadi, Muhammad Ashraf, Tayaba Ismail, Sohail Anjum Shahzad, Zulfiqar Ali Khan, Syed Ali Raza Naqvi, Nasir Mahmood

**Affiliations:** a Interdisciplinary Research Center in Biomedical Materials, COMSATS Institute of Information Technology, Lahore, 54000, Pakistan; b Faculty of Chemistry, University of Warsaw, 02-093 Warsaw, Pasteura 1, Poland and Department of Physicochemical Drug Analysis, Faculty of Pharmacy, Jagiellonian University Medical College, 30-688 Cracow, Medyczna 9, Poland; c Department of Chemistry, Government College University, Lahore, 54000, Pakistan; d Department of Chemistry, King Fahd University of Petroleum and Minerals, Dhahran, 31261, Saudi Arabia; e Department of Biochemistry & Biotechnology, The Islamia University of Bahawalpur, Bahawalpur, 63100, Pakistan; f Department of Chemistry, COMSATS Institute of Information Technology, Abbottabad, 22060, Pakistan; g Department of Chemistry, Government College University, Faisalabad, 38000, Pakistan; h Department of Allied Sciences and Chemical Pathology, University of Health Sciences, Lahore, 54600, Pakistan

**Keywords:** Acetylcholinesterase, Alzheimer’s disease, Butyrylcholinesterase, Hydrazides, Indole derivatives, Molecular docking, SAR.

## Abstract

Cholinesterases (ChEs) play a vital role in the regulation of cholinergic transmission. The inhibition of ChEs is considered to be involved in increasing acetylcholine level in the brain and thus has been implicated in the treatment of Alzheimer’s disease. We have designed and synthesized a series of novel indole derivatives and screened them for inhibition of acetylcholinesterase (AChE) and butyrylcholinesterase (BChE). Most of the tested compounds exhibited inhibitory activity against AChE and BChE. Among them **4f** and **6e** showed the highest AChE inhibitory activity with IC_50_ 91.21±0.06 and 68.52±0.04 μM, respectively. However compound **5a** exhibited the highest inhibitory activity against BChE (IC_50_ 55.21±0.12 μM).

## INTRODUCTION

Alzheimer’s disease (AD), the most common form of neurodegenerative senile dementia, is associated with selective loss of cholinergic neurons and reduced level of acetylcholine neurotransmitter. The illness is characterized by memory deficit and progressive impairment of cognitive functions [1]. It has been revealed that an estimated 35.6 million people worldwide live with dementia [2]. The cholinergic hypothesis postulates that Alzheimer’s is caused by a decrease in acetylcholine (ACh) level in the brain, leading to gradual neurodegeneration. In normal brain signaling, ACh, in turn, is related to preserving and accessing memory, as well as function [3]. 

Therefore, the mainstays of current pharmacotherapy of AD are drugs aimed at increasing the acetylcholine level through the inhibition of enzymes: acetylcholinesterase and butyrylcholinesterase [4-6]. Studies have shown that AChE performs secondary non-cholinergic functions and co-localizes with the β-amyloid peptide (Aβ) deposits present in the brain of Alzheimer’s patients. It has been postulated that due to the presence of peripheral anionic site (PAS), AChE may bind amyloid fibrils, stabilize them and induce a conformational transition from Aβ into its amyloidogenic form [7, 8].

While BChE is primarily found in plasma, liver, and muscle tissues, its biological function is not fully known. However, whereas AChE preferentially hydrolyzes acetyl esters such as acetylcholine, BChE hydrolyzes butyrylcholine [9-11].

**Fig. (1):**
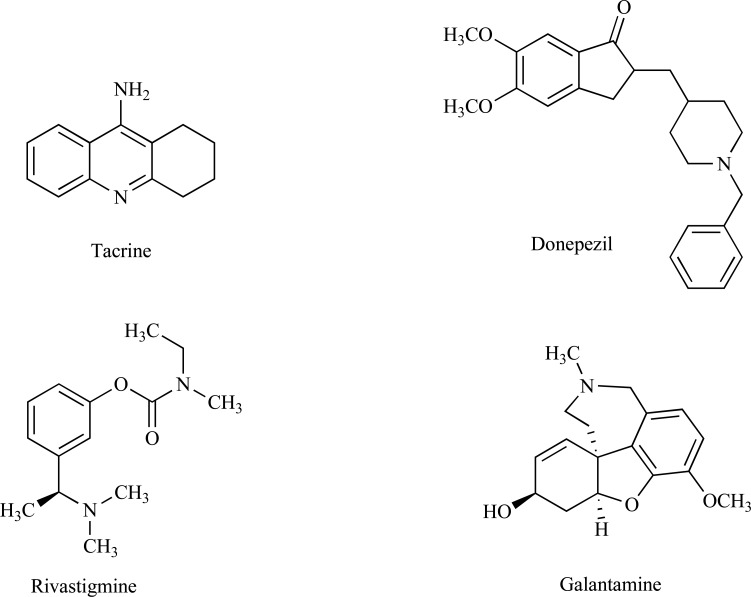
Chemical structures of the best known AChE inhibitors.

In order to understand the molecular pathogenesis of AD, enormous research efforts have been devoted in the past two decades [12]. The current therapeutic approach exploits the enhancement of the central cholinergic function [13] to increase the acetylcholine levels in the brain. As a result, various cholinergic drugs, such as tacrine, [14] donepezil, [15] rivastigmine, [16] and more recently galantamine [17] have been developed to alleviate the symptoms of AD (Fig. **1**). However, due to adverse events, tacrine was discontinued [18].

The potential effectiveness offered by the above inhibitors, unfortunately, is often limited by the appearance of central and peripheral side effects. For example, clinical studies have shown that tacrine has hepatotoxic liability [19, 20]. Therefore, large diversity of multi-target directed AChE inhibitors such as tacrine and nimodipine hybrids have also been evaluated [21, 22]. In addition, no therapeutic treatment is available for AD in Down syndrome [23].

**Fig. (2):**
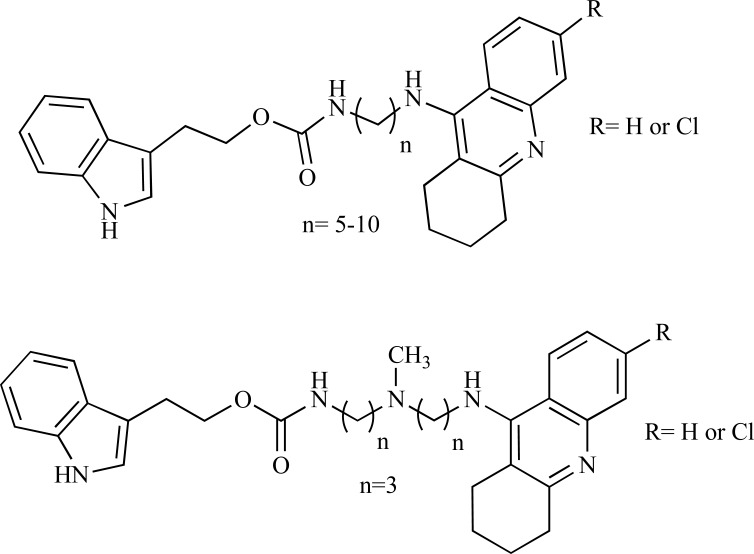
Indole group containing dual binding site AChE inhibitors.

Indole alkaloids are well known due to their wide biological importance [24, 25] such as inhibitors of AChE [26-28] and BChE [29, 30]. Compounds containing indole ring were also found to be the dual binding site AChE inhibitors (Fig. **2**), which in turn has a potential of disease modifying agents by inhibiting the Aβ peptide due to their binding ability with both the catalytic and peripheral sites of the enzymes [28]. Thus there is a great deal of interest in the development of dual binding site AChE inhibitors in order to control AD [26]. 

Based on the promising nature of the indole analogues, we have designed and synthesized a series of new indole-moiety containing compounds (**5a**-**5c**, **6a**-**6e**) along with known (**4a**-**4g**) and screened them for inhibition of AChE and BChE. This communication deals with the synthesis of these compounds and their biological and docking studies.

## MATERIALS AND METHODS

### Chemistry

**Scheme (1):**
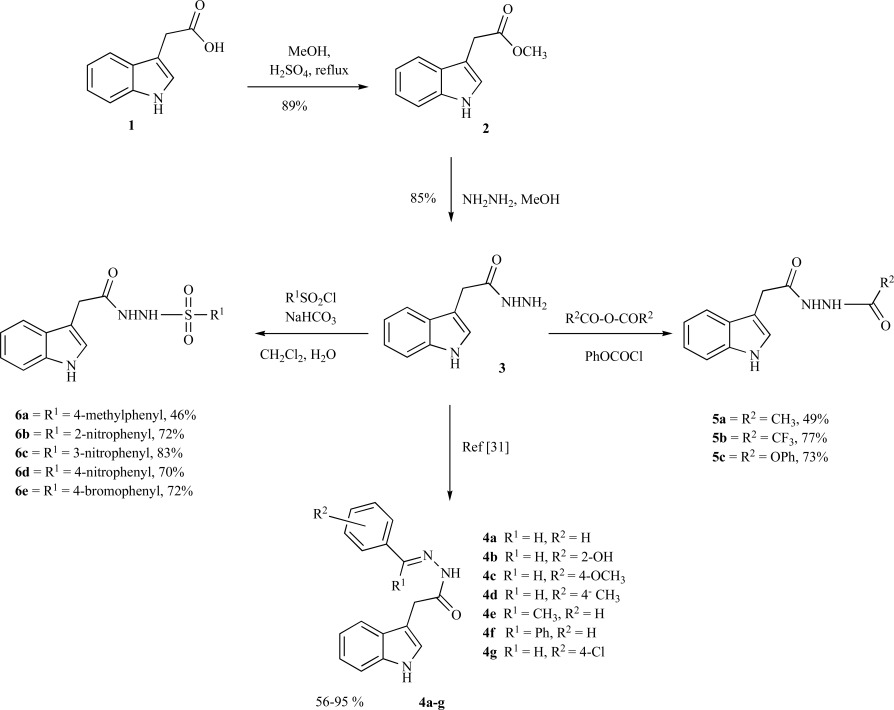
Synthetic protocol of indole derivatives.

Indole analogues **2-6** were synthesized from commercially available indole-3-acetic acid **1 **as depicted in Scheme **1**. Acid catalyzed esterification of **1** in methanol gave ester **2**, which was treated with hydrazine to produce the desired indole hydrazide **3** in 85% yield. Reaction of hydrazide **3** with a variety of sulfonyl chlorides in a mixture of dichloromethane and water produced the desired sulfonohydrazides **6a-6e**. Likewise, hydrazide **3** was condensed with acetic anhydride and trifluoroacetic anhydride to synthesize acetohydrazides **5a** and **5b** respectively (Scheme **1**). Similarly the hydrazine carboxylate **5c** was obtained by the reaction of phenyl chloroformate with hydrazide **3** in 73% yields (Scheme **1**). Compounds (**4a-4g**) were synthesized according to the literature procedures [31]. The structures of all new compounds were established with the aid of IR, ^1^H-NMR, ^13^C-NMR, mass spectrometry and elemental analyses.

### Material and Instruments

Reagents were purchased from common commercial suppliers and were used without further puriﬁcation. Solvents were puriﬁed and dried by standard procedures, when necessary. TLC was performed on silica coated aluminum plates (6F254, 0.2 mm). ^1^H-NMR and ^13^C-NMR spectra were recorded on Bruker NMR 500 MHz and chemical shifts were calculated with reference to CDCl_3_ (7.26). IR spectra were recorded on a Jasco A-302 IR spectrophotometer. Mass spectra were recorded on a Varian MAT 312 double focusing spectrometer, connected to an IBM-AT compatible PC computer system. Elemental analyses were recorded on the Elementar, Vario micro cube, Germany.

#### General Procedure (GP-1) for the Synthesis of (6a-6e)

To a solution of compound 3 (0.2 g, 1.06 mM) in 3 M aqueous NaHCO_3_ (2 mL) a solution of the corresponding sulfonyl chloride (1.16 mM) was added dropwise in 2 mL CH_2_Cl_2_ and the reaction mixture was stirred at room temperature for 4 hours. The precipitated product formed was filtered and successively washed with dilute HCl and n-hexane. The residue was puriﬁed by recrystallization in methanol to provide pure **6a**-**6e**.

#### N'-(2-(1H-Indol-3-yl)acetyl)-4-methylbenzenesulfonohydrazide (6a)

Following the general procedure (GP-1) the compound **6a** was obtained as a yellowish brown solid, yield 46%; m.p. 90 °C; R*f* (EtOAc: hexane, 2:1) 0.62; IR (KBr) cm^-1^ 3409 (NH), 3211, 3208 (NHNH), 1657 (C=O); ^1^H NMR (500 MHz; CD_3_OD): δ 7.71-6.86 (9H, m, Ar-H), 3.78 (2H, s, CH_2_), 2.35 (3H, s, CH_3_); ^13^C NMR (125 MHz; CD_3_OD): δ 172.7 (C=O), 145.4 (C), 142 (C), 138.2 (C), 136 (C), 130.4 (CH), 130.2 (CH), 130.1 (CH), 129.5 (CH), 128.8 (CH), 127.2 (CH), 125.1 (CH), 122.8 (CH), 120.1 (CH), 112.5 (C), 31.9 (CH_2_), 21.74 (CH_3_); MS m/z (%) 343 (M^+^); Anal. calc. for C_17_H_17_N_3_O_3_S: C, 59.46; H, 4.99; N, 12.24; found: C, C, 59.42; H, 4.91; N, 12.19.

#### N'-(2-(1H-Indol-3-yl)acetyl)-2-nitrobenzenesulfonohydrazide (6b)

Following the general procedure compound **6b** was obtained as an off-white solid; yield 72%; m.p. 254 °C; R*f* (EtOAc) 0.413; IR (KBr) cm^-1 ^3400 (NH), 3201, 3206 (NHNH), 1661 (C=O); ^1^H NMR (500 MHz; CD_3_OD): δ 7.9-6.9 (9H, m, ArH), 3.61 (2H, s, CH_2_); ^13^C NMR (125 MHz; CD_3_OD): δ 174.2 (C=O), 138.3 (C), 128.7 (C), 125.13 (CH), 122.8 (CH), 122.2 (CH), 121 (CH), 119.6 (CH), 112.5 (CH), 109.3 (C), 32.2 (CH_2_); MS m/z (%) 374 (M^+^); Anal. calc. for C_16_H_14_N_4_O_5_S: C, 51.33; H, 3.77; N, 14.97; found: C, 51.30; H, 3.71; N, 14.95.

#### N'-(2-(1H-Indol-3-yl)acetyl)-3-nitrobenzenesulfonohydrazide (6c)

Following the general procedure compound **6c** was obtained as golden yellow solid; yield 83%; m.p. > 350 °C; R*f* (EtOAc: hexane, 7:3) 0.282; IR: (KBr) cm^-1 ^3395 (NH), 3198, 3202 (NHNH), 1649 (C=O); ^1^H NMR(500 MHz; CD_3_OD): δ 8.62-7.01 (9H, m, ArH), 3.65 (2H, s, CH_2_); ^13^C NMR (125 MHz; CD_3_OD): δ 133.2 (CH), 131.2 (CH), 126 (CH), 122.2 (CH), 119.6 (CH), 33.8 (CH_2_); MS m/z (%) 374 (M^+^); Anal. calc. for C_16_H_14_N_4_O_5_S: C, 51.33; H, 3.77; N, 14.97; found: C, 51.29; H, 3.73; N, 14.91.

#### N'-(2-(1H-Indol-3-yl)acetyl)-4-nitrobenzenesulfonohydrazide (6d)

Following the general procedure (GP-1) the compound **6d** was obtained as a light yellow solid; yield 70%; m.p. 108 °C; R*f* (EtOAc) 0.739; IR (KBr) cm^-1 ^3403 (NH), 3225, 3219 (NHNH), 1655 (C=O); ^1^H NMR (500 MHz; CD_3_OD): δ 8.2-6.98 (9H, m, ArH), 3.65 (2H, s, CH2); ^13^C NMR (125 MHz; CD_3_OD): δ 172.8 (C=O), 150.4 (C), 131.6 (C), 130.7 (C), 128.6 (C), 125.3 (CH), 124.9 (CH), 124.6 (CH), 123 (CH), 120.2 (CH), 120 (CH), 112.7 (C), 32 (CH_2_); MS m/z (EI) 374; Anal. calc. for C_16_H_14_N_4_O_5_S: C, 51.33; H, 3.77; N, 14.97; found: C, 51.31; H, 3.76; N, 14.94.

#### N'-(2-(1H-indol-3-yl)acetyl)-4-bromobenzenesulfonohydrazide (6e)

Following the general procedure (GP-1) compound **6e** was obtained as light yellow crystalline solid; yield 72 %; m.p. 88 °C; Rf (EtOAc) 0.869; IR (KBr) cm^-1 ^3415 (NH), 3217, 3213 (NHNH), 1646 (C=O); ^1^H NMR (500 MHz; CD_3_OD): δ 7.73-6.9 (9H, m, ArH) 3.62 (2H, s, CH_2_); ^13^C NMR (125 MHz; CD_3_OD): δ 132.7 (C), 132.4 (C), 130.2 (C), 127.5 (CH), 125.1 (CH), 122.8 (CH), 120.2 (CH), 119.8 (CH), 112.5 (C), 32.8 (CH_2_); MS m/z (%) 406 (M^+^); Anal. calc. for C_16_H_14_BrN_3_O_3_S: C, 47.07; H, 3.46; Br, N, 10.29; found: C, 47.03; H, 3.42; Br, N, 10.26.

#### N'-acetyl-2-(1H-indol-3-yl)acetohydrazide (5a)

To a solution of compound **3** (0.2 g, 1.06 mM) in H_2_O (1.6 mL) acetic anhydride (0.1 mL, 1.16 mM) was added and the mixture was stirred for 2 hours at room temperature. The precipitated product was filtered off and washed with dilute HCl to remove unreactive hydrazide. Crystallization from methanol yielded **5a** as purple crystalline solid (0.12 g, 49%). m.p 117 °C; R*f* (EtOAc: hexane, 1:1) 0.36; IR (KBr) cm^-1^ 3401 (NH), 3191, 3188 (NHNH), 1633, 1666 (C=O); ^1^H NMR (500 MHz; CD_3_OD): δ 7.59-6.9 ( 5H, m, ArH), 3.7 (2H, s, CH_2_), 1.9 (3H, s, CH_3_); ^13^C NMR (125 MHz; CD_3_OD): δ 173.7 (C=O), 172.1 (C=O), 138 (C), 128.5 (C), 124.9 (CH), 122.5 (CH), 119.8 (CH), 119.4 (CH), 112.2 (CH), 108.7 (C), 31.8 (CH_2_), 20.4 (CH_3_); MS m/z (%) 231 (M^+^); Anal. calc. for C_13_H_15_N_2_O_2_: C, 62.33; H, 5.67; N, 18.17; found: C, 62.32; H, 5.63; N, 18.12.

#### N'-(2-(1H-indol-3-yl)acetyl)-2,2,2-trifluoroacetohydrazide (5b)

To a solution of compound **3** (0.2 g, 1.058 mM) in THF (5 mL) trifluoroacetic anhydride (0.2 mL, 1.164 mM) was added dropwise and the mixture was stirred at room temperature for 2 hours. The precipitated product was filtered off and washed successively with NaHCO_3_ (3 M) and diluted to obtain **5b** (0.23 g, 77%) as light brown solid; m.p. 120 °C; R*f* (EtOAc) 0.74; IR (KBr) cm^-1^ 3399 (NH), 3205, 3201 (NHNH), 1644, 1670 (C=O); ^1^H NMR (500 MHz; CD_3_OD): δ 7.76-6.99 (5H, m, ArH), 3.72 (2H, s, CH_2_); ^13^C NMR (125 MHz; CD_3_OD): δ 174.2 (C=O), 169.73 (C=O), 138.2 (C), 128.7 (C), 125.1 (CH), 122.7 (CH), 120.3 (CH), 119.6 (CH), 112.5 (CH), 109.2 (C), 32.26 (CH_2_); MS m/z (%) 285 (M^+^); Anal. calc. for C_12_H_10_F_3_N_3_O_2_: C, 50.53; H, 3.53; N, 14.73; found: C, 50.49; H, 3.51; F, 19.98; N, 14.69.

#### Phenyl 2-(2-(1H-indol-3-yl)acetyl)hydrazinecarboxylate (5c)

To a solution of compound **3** (0.2 g, 1.058 mM) in aqueous NaHCO_3_ (3 M, 1 mL) phenyl chloroformate (0.13 mL, 1.058 mM) was added dropwise and the mixture was stirred at room temperature for 2 hours. The precipitated product was filtered off and washed with dilute HCl to obtain **5c** (0.24 g, 73%) as a white solid. m.p. 148°C; R*f* (EtOAc) 0.64; IR: (KBr) cm^-1 ^3424 (NH), 3213, 3206 (NHNH), 1652, 1689 (C=O); ^1^H NMR (500 MHz; CD_3_OD): δ 7.7-6.9 (10H, m, ArH), 3.72 (2H, s, CH_2_); ^13^C NMR (125 MHz; CD_3_OD): δ 174.7 (C=O), 157.1 (C=O), 152.5 (C), 138.3 (C), 130.9 (CH), 130.7 (CH), 128.8 (C), 127.0 (CH), 126.9 (CH),125.1 (CH), 122.9 (CH), 122.8 (CH), 120.2 (CH), 119.7 (CH), 112.4 (CH), 108.9 (C), 32.1 (CH_2_); MS m/z (%) 309 (M^+^); Anal. calc. for C_17_H_15_N_3_O_3_: C, 66.01; H, 4.89; N, 13.58; found: C, 65.98; H, 4.83; N, 13.55.

### AChE and BChE Assay

The AChE and BChE inhibition activity was performed according to the method of Ellman [32] with slight modifications. Total volume of the reaction mixture was 100 µL contained 60 µL Na_2_HPO_4 _buffer with concentration of 50 mM and pH 7.7. A 10 µL test compound (0.5 mM per well) was added, followed by the addition of 10 µL enzyme (0.005 unit AChE, 0.5 unit BChE per well, Sigma Inc). The contents were mixed and pre-read at 405 nm and pre-incubated for 10 min at 37°C. The reaction was initiated by the addition of 10 µL of 0.5 mM per well substrate (acetylthiocholine iodide or butyrylthiocholine bromide), followed by the addition of 10 µL DTNB (0.5 mM per well). After 30 min of incubation at 37ºC, absorbance was measured at 405 nm. Synergy HT (BioTek, USA) 96-well plate reader was used in all experiments. All experiments were carried out with their respective controls in triplicate. Eserine (0.5 mM per well) was used as a positive control [33]. The percent inhibition was calculated by the help of following equation.

$$\mathrm{Inhibition}\left(\%\right)=\frac{\mathrm{Control}-\mathrm{Test}}{\mathrm{Control}}\times 100$$

IC_50_ values (concentration at which there is 50% enzyme inhibition) of compounds were calculated using EZ–Fit Enzyme kinetics software (Perella Scientific Inc. Amherst, USA).

### Docking Studies

Three-dimensional representation of ligand was created using Corina online tool [34] and saved as pdb file. Using Sybyl 8.0 [35] Gasteiger-Marsili charges were assigned following check of atom types and protonation of the compounds. Finally, ligand structure was saved in the mol2 format. Docking was performed to *Torpedo californica *AChE from 1EVE crystal complex [36] using Gold 5.1 program [37]. In the preparatory phase, all histidine residues were protonated at Nε, hydrogen atoms added, ligand molecules removed, and binding site defined as all amino acid residues within 10 Å from donepezil. The presence of some water molecules was also taken into account. A standard set of genetic algorithm with population size 100, number of operations 100 000 and clustering tolerance of 1 Å was applied. As a result, 20 ligand conformations were obtained and sorted according to ChemScore function values. Results were visualized by PyMOL [38].

## CONCLUSION

The synthesized compounds were screened for their inhibitory effect against AChE and BChE. Eserine was used as control [33] with inhibition percentage of 91.29±1.17 at the concentration of 0.5 mM. Among the series, compound **6e** was found to be the most active against AChE with an IC_50 _value of 68.52±0.04 µM (Table **1**). The seemingly preferred interaction of *p*-bromo benzene sulfonyl group of **6e **with enzyme may arise due to the planner orientation and polarity of bromo group. Compounds **4f** (IC_50_ 91.21±0.06 µM) and **4h** (IC_50_ 93.61±0.15 µM) also showed good activity, which could be attributed to the presence of more rigid structures and bulky biphenyl and naphthyl groups, which in turn may have caused greater hydrophobic interactions with enzyme and hence resulted in comparatively more inhibition. 

Likewise compounds **6c**, **5c**, **6b** and **6d **showed weak against AChE (Table **1**). The lower inhibition in case of **6b-6d** is considered to be due to the presence of much polar and bulky nitro group, which may have prevented effective interaction with the enzyme. However, in case of **5c **carbamate moiety might have played role in its lower inhibition. The overall order of inhibition against AChE was found to be:

**6e > 4f > 4h > 4d > 6a > 4g = 5b = 4b = 4c >2 > 3 > 4e > 4a > 5a > 6d >6c > 5c**

In case of BChE inhibition studies, compounds **5a**, **4f** and **5b** were the most active with an IC_50_ values of 55.21±0.12, 59.81±0.06 and 68.91±0.07 µM, respectively. In case of compounds **5a** and **5b**, an amide moiety might have played a role by providing more rigid structures which in return enhanced inhibitory power of these compounds. Compound **5a** was found to be slightly more active than **5b** which may be related to the size and electronic factors of acetyl vs trifluoroacetyl group. In addition, higher similarity of compound **5a** with acetylcholine compared to **5b** could also have played a role in its higher inhibition. The higher activity of **4f** could also be attributed due to the presence of biphenyl ring. Similarly, compounds **6b, 5c, 4d **and** 4b** showed weak inhibitory activities against BChE enzyme (Table **1**). 

The overall order of inhibition percentage against BChE was:

**5a > 4f > 5b >6c = 6e> 4a> 3 > 6a> 4e > 6d > 2 > 4c**

In the case of AChE inhibition, it was revealed that the chlorine substitution in the aromatic ring (**4g**) sharply enhanced the inhibition (**4a** *vs* **4g**, Table **1**). Likewise the size and hydrophobicity of the substituent also played a significant role; **4h** is a better inhibitor than **4b**. Similarly, electron releasing groups (OH, OCH_3_) in the aromatic ring also enhanced the inhibitory effect, for instance **4b** and **4c **were more active compared to **4a** (Table **1**). These groups help in to form hydrogen bonding which is quite important for binding with enzyme.

**Fig. (3):**
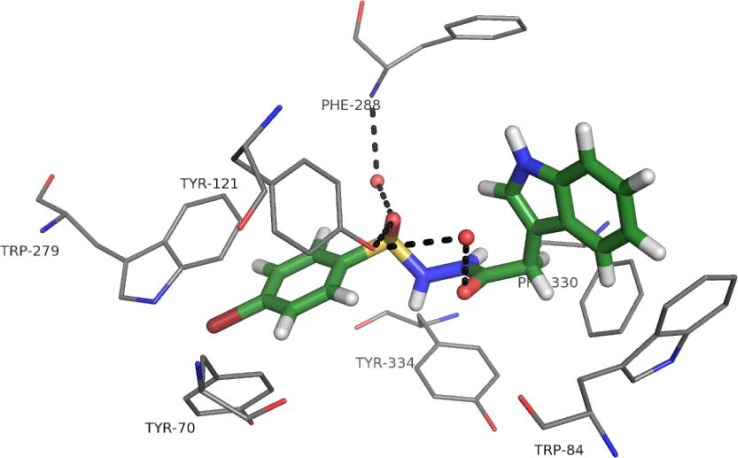
Binding mode of compound **6e** with AChE.

Among the whole series of indole derivatives, compound **6e** was selected for molecular docking studies. This compound showed the highest activity against AChE and even though its IC_50_ value was in the middle micro-molar range it was a good starting point for analysis. It was docked to the active gorge of AChE to find possible binding mode and to explain why activity was not high enough. In the second step it was possible to propose structural modifications which could improve the potency of novel derivatives. The AChE from 1EVE complex [36] was chosen as target structure according to the validation process, described elsewhere [39]. Among reference inhibitors from PDB complexes, donepezil was the most similar to novel compounds - they were linear molecules with two aromatic groups at the ends. This confirmed that 1EVE structure was a good choice. Docking studies revealed that compound **6e** was bound to both catalytic and peripheral active site (Fig. **3**). It is quite important because dual binding site derivatives can increase cholinergic transmission and inhibit AChE-dependent β-amyloid aggregation. The strength of binding was assessed by ChemScore function which adopted value 38.76 for ligand **6e** in comparison with 49.48 for reference compound - donepezil. It remained in accordance with experimental results because anti-AChE activity of donepezil is much higher than potency of compound **6e**. The IC_50_ values for reference and novel ligand were equal to 31.2 nM [39] and 68.52 µM, respectively. Derivative **6e** occurred in conformation with slightly bent linker. The outermost fragments of molecule interacted with two tryptophan residues: indole created CH-π interactions with Trp84, and *p-*bromophenyl formed π-π stacking interactions with Trp279. The chain should have been a bit longer to provide better fit to both tryptophan residues. The tether was engaged in H-bond network due to the presence of sulfonamide and amide fragments. One of the oxygen atoms from -SO_2_- group created hydrogen bond with hydroxyl group of Tyr121. The second one was a part of H-bond network: S=O ·····H_2_O (WAT1254) ·····HN-Phe288, and the carbonyl group formed the following bridge: C=O ·····H_2_O (WAT1159) ·····OH-Phe121 but its geometry was poor. It has seemed that introduction of one or two methylene groups between nitrogen atoms in the linker could improve the quality of that bridge and a fit of indole moiety to Trp84, leading to classical π-π stacking interaction.

In summary, the tested indole derivatives exhibited significant to good AChE and BChE inhibition. It has been observed that the nature and size of substituents have great influence on the activities of respective compounds. Compounds **6e** and the **5a** turned out to be the most active against AChE and BChE, respectively. These compounds may serve as a starting point in the discovery of cholinesterase inhibitors. Docking studies performed with **6e** has confirmed it as a dual binding site derivative. The strength of binding was assessed by ChemScore function which had value 38.76 for ligand **6e**. These observations strongly suggest a promise for the future drug discovery against AD. While we have demonstrated the importance of C-3 side chain in the inhibition of AChE and BChE, we do know that other features of the ligand can also further improve the inhibitory activities.

## Figures and Tables

**Table 1.** *In vitro* AChE & BChE Inhibition Activity of Compound 2-6e (Inhibition Percentage and IC50 Values are Means Given with SEM).

Entry	Compound	AChE Inhibition		BChE Inhibition	
		Inhibition (%) at 0.5 mM	IC_50_(µM)	Inhibition (%) at 0.5 mM	IC_50_(µM)
**1**	**2**	70.61±0.34	138.51±0.21	59.82±0.15	184.21±0.21
**2**	**3**	69.16±0.25	152.11±0.07	73.95±0.67	139.21±0.07
**3**	**4a**	63.11±0.52	198.61±0.14	79.58±0.42	86.31±0.14
**4**	**4b**	75.50±0.33	103.11±0.05	43.16±0.18	-
**5**	**4c**	75.50±0.56	109.61±0.11	50.22±0.69	<300
**6**	**4d**	79.54±0.54	98.81±0.14	43.61±0.55	-
**7**	**4e**	66.86±0.36	174.51±0.11	68.43±0.18	146.21±0.08
**8**	**4f**	89.34±0.91	91.21±0.06	89.51±0.69	59.81±0.06
**9**	**4g**	75.79±0.25	106.51±0.07	30.13±0.58	-
**10**	**5a**	60.52±0.25	298.61±0.15	90.81±0.69	55.21±0.12
**11**	**5b**	75.22±0.41	152.31±0.07	86.64±0.24	68.91±0.07
**12**	**5c**	55.91±0.33	<400	39.29±0.25	-
**13**	**6a**	78.11±0.36	121.41±0.16	72.52±0.33	101.21±0.16
**14**	**6b**	46.41±0.21	-	42.61±0.42	-
**15**	**6c**	56.21±0.25	<400	81.24±0.52	78.51±0.21
**16**	**6d**	58.62±0.52	<400	63.69±0.61	<400
**17**	**6e**	90.78±0.32	68.52±0.04	81.02±0.14	73.52±0.04
**Control**	**Eserine**	91.29±1.17	0.04±0.0001	82.82±1.09	0.85±0.0001
